# Quality of Life Following Traumatic Brain Injury in Iraqi Patients

**DOI:** 10.4314/ejhs.v34i2.6

**Published:** 2024-03

**Authors:** Ula Munther Al-Azzaw, Laith Thamer Al-Ameri

**Affiliations:** 1 Al-Kindy College of Medicine, University of Baghdad, Baghdad, Iraq

**Keywords:** satisfaction, brain trauma, QOLIBRI scale

## Abstract

**Background:**

Knowledge about quality of life following brain trauma is necessary to improve public health programmes.

**Methods:**

A cross-sectional study was conducted at Ghazi al-Hariri Surgical Specialties Hospital, Dr. Saad Al-Witry Neuroscience Hospital in the Baghdad governorate, and Baqubah General Hospital in the Diyala governorate from first January to the end of April 2022. The study's target population was patients aged 18 years and older with traumatic brain injury six to 12 months post-injury, Quality of life was evaluated by a structured questionnaire using the quality of life after brain injury (QOLIBRI) scale

**Results:**

A total of 225 participants were included. The highest proportion (52.9%) was within the age group of 18-29 years. The male-to-female ratio was 3.09:1. Road traffic accidents were the main cause of brain damage, affecting 67.6% and 52.7%, respectively. The average levels of satisfaction with thinking ability, emotions, independence, and social relationships were 56.9%, 52.9%, 42.2% and 43.6%, respectively. The average levels of dissatisfaction with feelings and physical activity were 48.4% and 53.8%, respectively.

**Conclusion:**

Most patients with traumatic brain injury had average overall satisfaction with their thinking abilities, emotions, independence, and social relationships. Majority of them had an average level of discontent with their feelings and a low level of discontent with their physical activity.

## Introduction

Globally, millions of people are affected by traumatic brain injury (TBI), the incidence of which varies from 60 cases per 100,000 to 12-fold (1, 2), with more than 50% mortality in severe TBI and even 80% in the elderly population (3,4). Surviving after TBI with a prolonged hospital stay may result in long-term physical, cognitive, sleep, and psychological disorders that may require prolonged rehabilitation (5, 6). These factors negatively impact previous relationships and prevent individuals from returning to work, and adversely impacting socioeconomic outcomes. Additionally, survivors have a relatively shorter life expectancy (7).

Knowledge about quality of life (QOL) following TBI is necessary to improve public health programmes and to implement protocols for its management. Additionally, it provides good medical care and rehabilitation services for patients aiming to improve outcomes and lessen the likelihood of developing cognitive and emotional consequences that are directly related to the severity of trauma and physical disability (7).

TBI may result from many mechanisms, including a blunt blow or a penetrating head injury that can affect brain function (8, 9). Additionally, blast injury is a principal cause of TBI in individuals in active military service during warfare (10). The TBI severity was divided into mild, moderate, and severe. TBI severity results in different signs and symptoms that may range from brief loss of consciousness to convulsions, coma, or death (11). The Glasgow Coma Scale (GCS) score is used to assess TBI severity (12, 13). Globally, 80% of reported head injuries are mild, 10% are moderate, and 10% are severe (14, 15).

QOL is defined as how a person evaluates the ‘goodness’ of many life features (16). Different scales are available to assess outcomes (17), including return to work (RTW). Nevertheless, a thorough instrument was required to reflect the objective and subjective aspects of patients' outcomes and to assess rehabilitation efforts. The quality of life after brain injury (QOLIBRI) was developed by the QOLIBRI Task Force (18-19). The QOLIBRI allows patients after TBI to self-rate their HRQOL (health-related quality of life) subjectively on six subscales (cognition, emotions and self-perception, daily life and autonomy, social relationships, emotions and negative feelings, and physical problems). The QOLIBRI has been validated, and its psychometric features have been evaluated in many regions and languages (18-21). The findings of the QOLIBRI searches suggest that the QOLIBRI is a unique and useful tool.

The current study aimed to evaluate QOL in Iraqi individuals with previous TBIs. Additionally, study aims to assess the possible correlations between QOL components and certain patient factors.

## Methods

This was a cross-sectional study conducted at Ghazi al-Hariri Surgical Specialties Hospital, Saad Al-Witry Neuroscience Hospital in the Baghdad governorate, and Baqubah General Hospital in the Diyala governorate. Study period running from the beginning of January to the end of April 2022.

Patients with a history of TBI in the previous 6-12 months, diagnosed according to Disease Control and Prevention (CDC) criteria, and 18 years and older were included in the study. Patients with an extended Glasgow Outcome Scale (GOSE) score less than 3; a spinal cord injury; significant other previous trauma; a psychiatric history; ongoing addiction; inability to understand, cooperate, or answer; or terminal illness were excluded.

QOL was evaluated by a structured questionnaire using the QOLIBRI scale (22), developed by an international taskforce. The researchers translated the questionnaire into Arabic and had it retranslated it into English by a third professional personnel to ensure its validity. The questionnaire was pretested through a pilot study among ten patients, who were not included in the study.

The questionnaire has two domains. The first domain included demographic characteristics; namely, age, sex, marital status, education, living arrangements, and job, with information about the duration since the injury, cause of injury, GCS score, and Glasgow Outcome Scale extended (GOSE) score. The GOSE scale contains five categories (dead, vegetative, severe disability, moderate disability, and good recovery). Only the last three categories out of the five were used for the GOSE score in this study. The second domain was the QOLIBRI scale (22), which consists of two parts. The first part consists of four sections about satisfaction with thinking abilities, emotions and self-image, independence, and social relationships, while the second part consists of two sections about how bothered the patient is about the currently developed feelings and physical problems.

For scoring, five possible answers were given for each question in the QOLIBRI scale: Not at all, Slightly, Moderately, Quite, and Very. The questions had five scores: 1, 2, 3, 4, and 5. The data were collected through direct interviews; patients were enrolled during their follow-up visits to the outpatient consultation clinic of the hospitals mentioned above.

Participation in the study was voluntary. The researchers explained the study's objectives to the patients and their relatives. Written consent was obtained from all patients who agreed to participate in the study. All the participants were given a complete unconditioned choice to participate in the study; they were allowed to withdraw at any time they felt uncomfortable. Complete confidentiality was ensured, all the collected data were used for research purposes only, and personal information was collected with serial identification numbers without an identity.

The study was implemented following approval by the scientific board at the Al-Kindy College of Medicine after discussing the proposal. Permission was obtained from the Ministry of Health, the Al-Rusafa Health Directorate, and the Diyala Health Directorate. A collaboration between Ghazi Al-Hariri Surgical Specialties Hospital, Dr. Saad Al-Witry Neuroscience Hospital in the Baghdad Governorate, and Baqubah General Hospital in the Diyala Governorate was ensured through official correspondence.

The data were analyzed with SPSS (Statistical program version 24). Parametric data are presented as the mean and standard deviation. Categorical data are presented as numbers and percentages. The chi-square test and Fisher exact test were used to test for homogeneity. Independent t tests and ANOVAs were used to analyze the differences between groups' parametric variables. A P value < 0.05 was considered to indicate statistical significance.

## Results

A total of 225 patients with previous brain injury were included. The highest proportion, 52.9% (119), was within the age group of 18-29 years. The male-to-female ratio was 3.09:1. There were 111(49.3%) married participants. The majority, 74(32.9%), had finished secondary education. Independents were the majority (n=183,81.3%). Most of them were unemployed (n=76,33.8%).

The mean age was 34 ± 15 years. The mean duration since brain injury was 9 ± 2 months. The mean GCS score was 8 ± 2. The mean GCS score was 6 ± 1, and the majority of traumatic brain injuries were caused by RTA (n=144, 64%). A total of 141(63%) patients enrolled in the study had less than nine months since brain injury. A total of 169 (75%) had severe injury with GCSs during injury 4-8.

Regarding satisfaction with thinking abilities, the most common answer was a moderate to six out of the seven questions. The majority of the participants responded moderately to six questions out of seven regarding satisfaction with emotions. The most frequent answer was moderately to four questions out of seven regarding satisfaction with their independence and function in daily life. The most frequent answer was “very” in four questions out of six regarding satisfaction with social relationships.

Regarding discontent with their feelings, the majority responded “very” to three questions out of five, whereas regarding discontent with physical activity, the majority responded moderately and not at all to two questions out of five.

**Figure 1:**
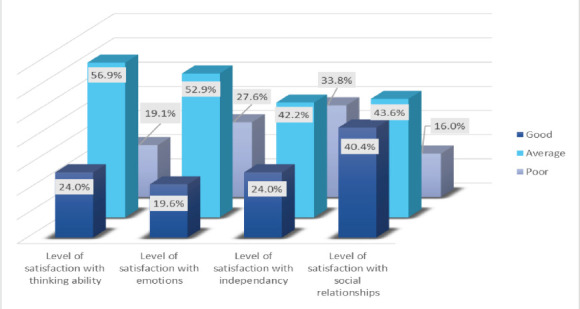
Patients level of satisfaction with different aspects after brain injury

**Figure 2:**
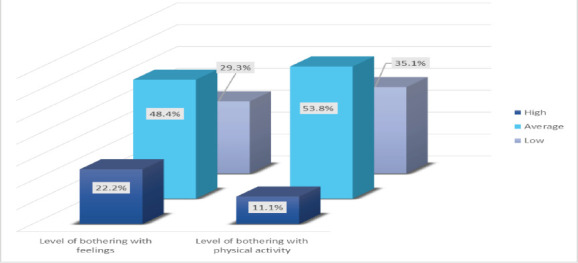
Patients level of bothering with different aspects after brain injury

One hundred twenty-eight participants were within an average level of satisfaction with their thinking ability. A total of 119 patients had an average level of satisfaction with their emotions. Additionally, 95 and 98 patients showed average satisfaction with independence and social relationships, respectively (Figure 1). In addition, 109 and 121 patients had an average level of discontent with their feelings and physical activity, respectively (Figure 2).

There was a significant association between duration since brain injury and satisfaction with thinking ability, where poor and average satisfaction were the most common factors [26 (60.5%) and 91 (71.1%), respectively], among those with a duration ≤ 9 months. However, good satisfaction was the highest among those with a duration > 9 months (P=0.003). There was a significant association between duration since brain injury and satisfaction with emotions, where poor and average satisfaction were distributed the most [49 (79%) and 73 (61.3%), respectively] among those with a duration ≤ 9 months. However, good satisfaction was the highest among those with a duration > 9 months (P=0.001).

As shown in Table 1, there was a significant association between duration since brain injury and satisfaction with independence, where poor and average satisfaction were the most common factors [52 (68.4%) and 64 (67.4%), respectively], among those with a duration ≤ 9 months. However, good satisfaction was the highest among those with a duration > 9 months (P=0.017). There was a significant association between duration since brain injury and satisfaction with social relationships, where poor and average satisfaction were the most common factors [25 (69.4%) and 68 (69.4%), respectively], among those with a duration ≤ 9 months. However, good satisfaction was the highest among those with a duration > 9 months (P=0.040). There were no significant associations between sex and level of satisfaction or discontent in patients with previous brain injury, as illustrated in Table 2.

**Table 1:** Distribution the level of satisfaction and bothering in relation with the time since brain injury

Scale		Total	Time since injury				P-value

			≤ 9 months		> 9 months		

			N.	%	N.	%	
**Level of satisfaction with thinking ability**	**Poor**	43	26	60.5	17	39.5	
	**Average**	128	91	71.1	37	28.9	0.003*
	**Good**	54	24	44.4	30	55.6	
**Level of satisfaction with emotions**	**Poor**	62	49	79.0	13	21.0	
	**Average**	119	73	61.3	46	38.7	0.001*
	**Good**	44	19	43.2	25	56.8	
**Level of satisfaction with independency**	**Poor**	76	52	68.4	24	31.6	
	**Average**	95	64	67.4	31	32.6	0.017*
	**Good**	54	25	46.3	29	53.7	
**Level of satisfaction with social relationships**	**Poor**	36	25	69.4	11	30.6	
	**Average**	98	68	69.4	30	30.6	0.040*
	**Good**	91	48	52.7	43	47.3	
**Level of bothering with feelings**	**Low**	66	38	57.6	28	42.4	
	**Average**	109	67	61.5	42	38.5	0.265
	**High**	50	36	72.0	14	28.0	
**Level of bothering with physical activity**	**Low**	79	48	60.8	31	39.2	
	**Average**	121	76	62.8	45	37.2	0.807
	**High**	25	17	68.0	8	32.0	

* Significant at P < 0.05.

**Table 2:** Distribution the level of satisfaction and bothering in relation with gender

Scale		Total	Gender				P-value

			Male		Female		

			N.	%	N.	%	
**Level of satisfaction with thinking ability**	**Poor**	43	35	81.4	8	18.6	
	**Average**	128	90	70.3	38	29.7	0.107
	**Good**	54	45	83.3	9	16.7	
**Level of satisfaction with emotions**	**Poor**	62	42	67.7	20	32.3	
	**Average**	119	90	75.6	29	24.4	0.089
	**Good**	44	38	86.4	6	13.6	
**Level of satisfaction with independency**	**Poor**	76	58	76.3	18	23.7	
	**Average**	95	69	72.6	26	27.4	0.622
	**Good**	54	43	79.6	11	20.4	
**Level of satisfaction with social relationships**	**Poor**	36	28	77.8	8	22.2	
	**Average**	98	71	72.4	27	27.6	0.635
	**Good**	91	71	78.0	20	22.0	
**Level of bothering with feelings**	**Low**	66	50	75.8	16	24.2	
	**Average**	109	80	73.4	29	26.6	0.666
	**High**	50	40	80.0	10	20.0	
**Level of bothering with physical activity**	**Low**	79	62	78.5	17	21.5	
	**Average**	121	92	76.0	29	24.0	0.335
	**High**	25	16	64.0	9	36.0	

A higher GOSE score was significantly associated with a higher level of satisfaction and a lower level of discontent for all the QOLIBI items (P= <0.001, <0.001, <0.001, 0.020, 0.010, and <0.001, respectively) (Table 3).

**Table 3:** Distribution the level of satisfaction and bothering in relation to GOSE

Scale		Total	GOSE				

			3-6		7-8		
			Severe and Moderate		Mild		P-value
	
			N.	%	N.	%	
**Level of satisfaction with thinking ability**	**Poor**	43	38	88.4	5	11.6	
	**Average**	128	86	67.2	42	32.8	<0.001*
	**Good**	54	22	40.7	32	59.3	
**Level of satisfaction with emotions**	**Poor**	62	49	79.0	13	21.0	
	**Average**	119	80	67.2	39	32.8	<0.001*
	**Good**	44	17	38.6	27	61.4	
**Level of satisfaction with independency**	**Poor**	76	72	94.7	4	5.3	
	**Average**	95	70	73.7	25	26.3	<0.001*
	**Good**	54	4	7.4	50	92.6	
**Level of satisfaction with social relationships**	**Poor**	36	24	66.7	12	33.3	
	**Average**	98	75	76.5	23	23.5	0.020*
	**Good**	91	47	51.6	44	48.4	
**Level of bothering with feelings**	**Low**	66	34	51.5	32	48.5	
	**Average**	109	73	67.0	36	33.0	0.010*
	**High**	50	39	78.0	11	22.0	
**Level of bothering with physical activity**	**Low**	79	27	34.2	52	65.8	
	**Average**	121	96	79.3	25	20.7	<0.001*
	**High**	25	23	92.0	2	8.0	

* Significant at P < 0.05

## Discussion

In the present study, the highest proportions of the participants were younger than 39 years; 75.6% of the participants were males, which is what has been reported in many studies (21,23,24,25,26), as males are more likely to engage in injury-prone work and dangerous behaviors (26). The majority of the patients had traumatic brain injury due to road traffic accidents (RTAs), which aligns with the findings of the previously mentioned studies (21, 23, 24, 25). The results also showed that the most common cause of TBI in males was RTA (67.6%), whereas RTA contributed to 52.7% of the total deaths in females. Male drivers, especially those on highways, were more common than female drivers were; tended to drive at higher speeds more than females did; and had less commitment to wearing safety belts than females did (27). Moreover, males using phones while driving carried a greater risk of distraction than females do according to the findings of previous studies (27, 28). This may explain why males are the most common victims of RTA and TBI.

Head physical trauma in females was double the prevalence in males. Ki Seong et al. (29) reported that, in their study conducted in South Korea in 2020, there was no significant difference in gender or cause of TBI or in RTA or physical trauma. However, the proportions of individuals struck on the head and assault were greater in males, while the proportions of pedestrians, slips and falls were greater in females.

Regarding satisfaction with thinking abilities, emotions, and independence, the majority of respondents responded moderately satisfied with the seven items in these domains. While they were satisfied with their social relationships, the majority of the participants responded “very much” satisfied with the six items in the domain. Moreover, the patients in the current study described their discontent with feelings with a “Very” in response to most of the five items in this domain while being unhappy with their physical activity.

In contrast, the majority responded to moderate and not at all in this domain. These findings in the current study might be explained by the findings of Milders et al. (30), in which, when investigating any impairment in patients' emotional and social function after head injury, they found that patients with TBI had significant emotional and social behavioral impairment compared to their relatives and a control group. Patients with TBI showed a significant increase in unusual and inappropriate behaviors that were not present before the injury.

TBI patients also had an increase in depression incidence, apathy, social withdrawal, and a decrease in communicative disabilities. In the Milders et al., they also measured emotional expression by face and voice and found a significant impairment in their emotional expression without impairment in empathy. Another study by Proctor et al. (31) was conducted to measure satisfaction with life after brain injury and the influences of social and psychological aspects on this satisfaction; they also reported different findings regarding the impact of TBI on quality of life, which supports our study. They used different scales for measuring life satisfaction, including the social isolation scale, leisure satisfaction scale, ten-item personality inventory, and satisfaction with life scale, and they found that TBI patients' life satisfaction did not improve as time passed since the injury. They also reported that community involvement and social activity positively affect life satisfaction in different aspects, including social relationships, emotions, and dependence, of TBI patients.

In the present study, the level of satisfaction was significantly associated with the time since injury, where a poor level of satisfaction (thinking abilities, emotions, independence, and social relationships) was greater among patients with less than nine months since the injury. A good level of satisfaction with those aspects was observed among patients with more than nine months since the injury. These findings are in line with those of a study by Scholten et al. (26). Their study used the Social Functioning 36-item SF-36 (version 1) and the Perceived Quality of Life Scale (PQoL) as measurements. This was a prospective cohort study in which TBI patients were treated at 6 and 12 months post-injury. They found that patient satisfaction improved over time after the injury. These authors explained that these findings can be attributed to improved physical functioning, reduced bodily pain, and gradual return to social functioning. Furthermore, this improvement over time included all the SF-36 domains, except for the mental health domain, which deteriorates over time for patients with severe TBI.

Regarding the differences in life satisfaction according to sex, the current study revealed no significant difference in the levels of satisfaction or discontent between male and female TBI patients. This agrees with the findings of other studies by Hawthorne et al. and Sagberg F. et al. (21, 32).

The present study revealed a significant association between the overall level of satisfaction in all the QOLIBR domains and the GOSE score. Specifically, poor levels of satisfaction in all the QOLIBR domains were significantly distributed, with the highest proportion of patients having GOSE scores from 4-6 (severe and moderate category). However, higher levels of satisfaction in all the QOLIBR domains were significantly distributed among patients with GOSE scores of 7-8 (good recovery category) (p-value for association of GOSE score and level of satisfaction with thinking abilities, emotions, independence, and social relationships = <0.001, <0.001, <0.001, and 0.020, respectively).

Patients with high levels of discontent were significantly distributed among patients with GOSE scores 4-6 (p-value for association of GOSE score and level of discontent with their feelings and physical activity = 0.010 and <0.001, respectively). These findings were in accordance with those of Scholten et al. (26), who reported a strong relationship between the GOSE score and SF-36 score (especially for physical functioning, P < 0.001; for social functioning, P < 0.001). The mean scores of all eight SF-36 domains were significantly greater in patients with higher GOSE scores at six months and 12 months post-injury. The PQoL also significantly increased with increasing GOSE score for patients of six months and 12 months post-injury. Another study by Mailhan et al. (33) explored TBI survivors' quality of life and assessed the relationship between life satisfaction and disability. The authors used the Subjective Quality of Life Profile (35 SQLP items) to measure quality of life and the GOSE as a measure of disability, and cognitive and behavioral impairment were assessed by the Neurobehavioral Rating Scale-Revised (NRS-R). They found a nonlinear relationship between life satisfaction and disability, where TBI patients with a GOSE score of 5-6 (moderate category) had the lowest satisfaction score, whereas the severe and good recovery categories had significant differences in life satisfaction.

The difference between our study and that of Mailhan et al. is that in our study, we combined the severe and moderate GOSE categories in one group and compared them with those in a good recovery group. In addition, Mailhan et al. studied TBI patients for more than two years.

The lack of significant differences in life satisfaction between TBI patients in the severe category and those in the good recovery category and between the moderate category might be attributed to the possibility that TBI patients in the severe category lack awareness of their cognitive and behavioral changes, increasing their satisfaction levels and scores in those two domains.

As a limitation, the current study assessed the quality of life of TBI patients for a period of 6-12 months post-injury. Additionally, patients with chronic medical conditions should be excluded or included in a stratified analysis because these comorbidities may be complicated by physical or neuropsychiatric conditions that may confound the relationship between quality of life and mental and functional outcomes in TBI patients. Future studies with the use of artificial intelligence (AI) are recommended for more chronic patients to handle and evaluate the massive amount of data. However, ethical concern surrounding AI should be approached mindfully (34).

In conclusion, most patients had average overall satisfaction with their thinking abilities, emotions, independence, and social relationships, while the majority had an average level of discontent with their feelings and a low level of discontent with their physical activity. Patients with higher GOSE scores had the highest levels of satisfaction and low levels of discontent. The longer the duration of TBI is, the better the patients' quality of life will be.
